# Heterogeneous connectivity can positively and negatively modulate the correlation between neural representations

**DOI:** 10.1186/1471-2202-14-S1-P391

**Published:** 2013-07-08

**Authors:** Wen-Chuang Chou, André Fiala, Marc Timme

**Affiliations:** 1 Network Dynamics, Max Planck Institute for Dynamics and Self-Organization, 37077 Göttingen, Germany; 2 Bernstein Center for Computational Neuroscience, 37073 Göttingen, Germany; 3 Molecular Neurobiology of Behavior, Johann-Friedrich-Blumenbach-Institute for Zoology and Anthropology, Georg-August-University of Göttingen, 37077 Göttingen, Germany

## 

**Figure 1:**
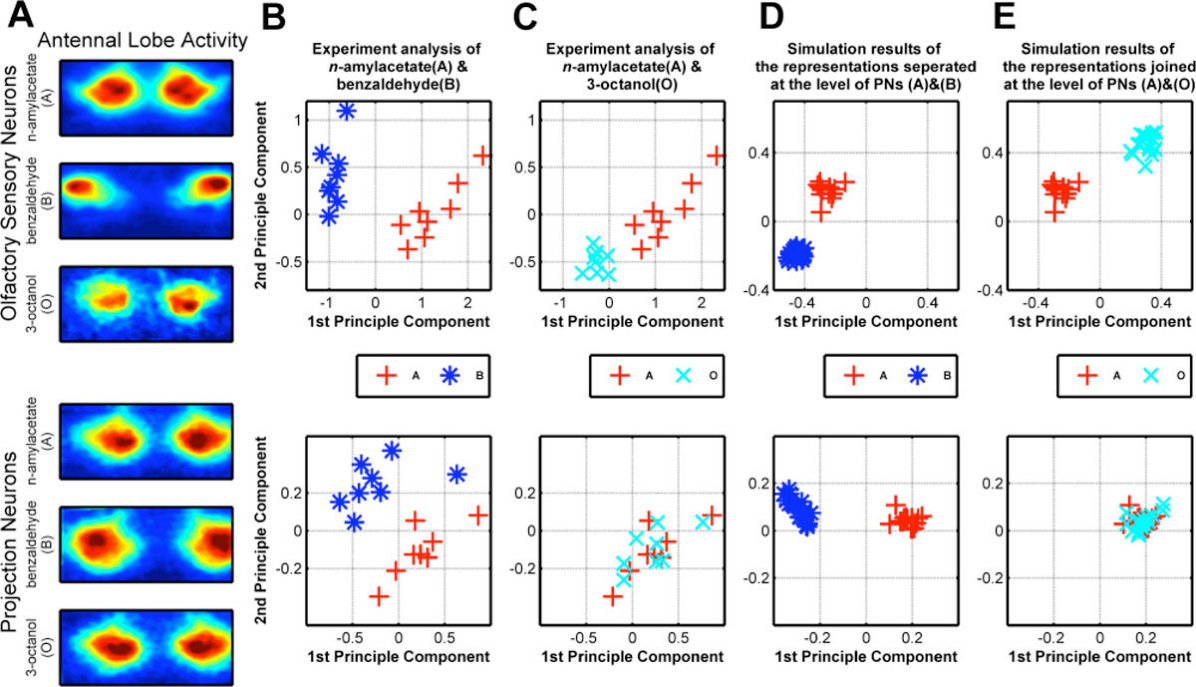
**Combining heterogeneous connectivity of and specific inhibition by local neurons may induce both joining and separating neural representations**.

To differentiate pooled external stimuli the sensory center requires distinct neural representations. It is generally considered that one major function of the antennal lobe in Drosophila is to separate the inputs from olfactory sensory neurons (SNs) and encode them into only weakly uncorrelated neural representations at the level of projection neurons (PNs). Several reports [1,2] demonstrated that the global dynamic of antennal lobe exhibits an inclination to separate odors and improve odor representations at the level of PNs. Our recent study showed that the sensory processing in the antennal lobe may also merge two odors in PNs although they are distinctly represented at the SN level [3]. Our experimental evidence suggests that the neural representations of the pair n-amylacetate (A) and benzaldehyde (B) are separated both at the SN and the PN levels (Figure 1A, B). Surprisingly, the representations of the pair n-amylacetate (A) and 3-octanol (O) are joined in the PNs although the input odorant-evoked activity patterns of the SNs are separated (Figure 1A, C); therefore, the correlation between that odor pair during the antennal lobe processing decreases. Such unexpected dynamics suggests that the function of antennal lobe should be reevaluated.

Here, we show that the correlation between odor representations can be either decreased or increased in a model of the antennal lobe given the identical heterogeneous glomerular innervation of local neurons (LNs). The investigation of glomerular innervation of LNs confirmed that whereas some LNs innervate to only a few glomeruli, others innervate many and yet others to almost all glomeruli [4]. Our simulation results showed that the different inputs of odorant-evoked activity patterns in SNs can be transformed into separated or joined PN representations of odor pairs in the identical circuits of LNs innervating all or part of glomeruli (Figure 1D, E). Our main contribution is grounded in the observation that selectively heterogeneous glomerular innervation of LNs may selectively inhibit particular sets of PNs, thereby reducing the high-dimensional inputs to lower dimensional activity of a glomerular circuit and thus modulating the dissimilarity or correlation between information encoded in PNs. Our analysis suggests that this connection heterogeneity may indeed be a key to resolve the conflicting functions enhancing the discriminating of odor representations (increasing correlation) and being robust against noise (decreasing the correlation between odor stimuli consisting of same odorants but being blurred by noise) in olfactory processing.
